# Network meta-analysis in psychology and educational sciences: A systematic review of their characteristics

**DOI:** 10.3758/s13428-022-01905-5

**Published:** 2022-07-11

**Authors:** Belén Fernández-Castilla, Wim Van den Noortgate

**Affiliations:** 1 ITEC, an imec research group at KU Leuven, Leuven, Belgium; 2 Faculty of Psychology and Educational Science, KU Leuven, Etienne Sabbelaan 51, 8500 Kortrijk, Belgium; 3 Universidad Nacional de Educación a Distancia, Madrid, Spain

**Keywords:** Systematic review, Network meta-analysis, Psychology and educational sciences

## Abstract

Network meta-analysis (NMA) allows the combination of evidence on the effectiveness of several interventions. NMA has mainly been applied in the medical science field, whereas in the domain of psychology and educational sciences its use is less frequent. Consequently, systematic reviews that describe the characteristics of published NMAs are limited to the field of medicine, and nothing is known about the characteristics of NMAs published in the psychology and educational sciences field. However, this information is still relevant for the design of future simulation studies and for detecting good and bad research practices. Thus, this study describes the features of the meta-analytic datasets of NMAs published in the field of psychology and educational sciences, as well as their methodological characteristics, and compares them to those observed in the medical domain. Results show that the number of studies included is larger in NMAs from psychology and educational sciences, the most commonly used effect size is the standardized mean difference (unlike the odds ratio in medicine), the sample size is smaller, more intervention groups are included, and inconsistent effects are observed more often. These results can be used in future simulation studies to generate realistic datasets. Finally, we warn about the poor quality of reporting of some technical aspects of the NMA, such as the statistical model used.

**Supplementary Information:**

The online version contains supplementary material available at 10.3758/s13428-022-01905-5.

One major goal in meta-analysis is to obtain more accurate conclusions about a given research question through the accumulation of evidence. When all the evidence existing on a given area is combined, results and conclusions are more reliable, accurate, and generalizable (Card, 2011). In a standard meta-analysis, direct evidence (expressed in effect sizes) is combined across studies. That is, if a researcher is interested in combining all the existing evidence on the effectiveness of treatment A relative to a control group, they will pool all the effect sizes from studies that address this comparison, and then conclude whether treatment A is actually effective. However, in psychology and educational sciences, typically several treatments/therapies/interventions exist to deal with a common problem (e.g., for treating panic disorder, several interventions can be applied, such as cognitive behavioral therapy, psychoeducation, or supportive psychotherapy; Pompoli et al., 2016), and researchers are often interested in knowing which of these interventions is the most effective. To this end, one approach is to carry out as many pairwise meta-analyses as potential comparisons between treatments exist. However, not only is this process time-consuming if there are many treatments, but it is also sometimes unfeasible, because it is common to find that two or more treatments have never been directly compared in primary research, leaving an important gap in this series of pairwise-comparisons. It is precisely at this point where network meta-analysis (NMA), also known as multiple-treatment comparison meta-analysis or as mixed-treatment comparison meta-analysis (Lumley, 2002; Salanti, Higgins, et al., 2008), emerges as a potentially useful methodology that allows us to estimate the indirect effects of pairs of treatments that have never been directly compared, and then to combine the direct and indirect evidence to increase the precision of the results and conclusions (Lumley, 2002; Lu & Ades, 2004).

## Rationale for the study

The use of NMA has increased substantially in recent years (Lee, 2013; Nikolakopoulou et al., 2014). A quick search in the Web of Science shows that in 2010, only 18 studies used the term “network meta-analysis,” whereas in 2018, 2019, and 2020, this number increased to 809, 1066, and 1188, respectively. If this search is disaggregated by discipline, we can see that this methodology has been mainly implemented in the medical sciences (Lee, 2013; Zarin et al., 2017), with 1034 studies using the term “network meta-analysis” in 2020. This contrasts with the number of studies that are found for the psychology and educational sciences domain: only 35 used the term “network meta-analysis” in 2020. Some studies have already noted the lack of use of NMA in the psychology and educational sciences field (e.g., Molloy et al., 2018), although in this area the comparison of the effectiveness of several competing interventions is also highly relevant.

Since most NMAs are published in the medical science field, systematic reviews that describe the characteristics of these NMAs are restricted to this domain (Chaimani et al., 2013; Lee, 2013; Nikolakopoulou et al., 2014; Petropoulou et al., 2016; Song et al., 2011b; Veroniki et al., 2013; Zarin et al., 2017), and little is known about the features of the NMAs published in the field of psychology and education. Differences between disciplines are expected: in NMAs within the medical science field, for instance, the most common effect size is the odds ratio (Petropoulou et al., 2016), whereas in psychology and educational sciences, the standardized mean difference is the most commonly used (Borenstein et al., 2009). Other examples of the differences between those fields are found in the systematic review by Fernández-Castilla et al. (2020), which showed for instance that the range of studies included in multilevel meta-analyses in the medical science field (6–88; interquartile range: 15–48) is more restricted than the range of studies included in behavioral and social sciences meta-analyses (5–456; interquartile range: 23–69). Describing the differences between the characteristics of NMAs in these fields is especially relevant for the design of future simulation studies that explore the performance of statistical methods to carry out NMA. In the existing simulation studies in NMA (e.g., Glenny et al., 2005; Kibret, 2013; Kiefer et al., 2020; Mills et al., 2011; Song et al., 2012; Veroniki et al., 2014), data were generated according to the characteristics described in systematic reviews of NMAs performed in the medical sciences domain. For instance, all these simulation studies generated odds ratio as effect sizes. If more differences are found between the characteristics of NMA in both fields, then the results of the existing simulation studies would be less informative for NMA in the field of psychology and education.

To address all of this, the main goal of this systematic review is to describe for the first time the characteristics of NMAs published in the domain of psychology and educational sciences Specifically, we will first describe the characteristics of these meta-analytic datasets, and we will compare them with those already described for meta-analytic datasets of NMAs published in the medical sciences. In this way, future simulation studies can use this information to generate realistic conditions that represent typical meta-analytic data from both domains. In addition, a second aim of this systematic review is to describe how behavioral and educational researchers carry out NMAs, that is, how they test the assumptions of this methodology (i.e., transitivity and consistency), the specific statistical models and software they use, and whether inconsistency effects are commonly found, among other characteristics. Also, this systematic review will provide information on whether these NMAs comply with the principles of reproducibility and transparency by making their datasets and code available (Klein et al., 2018). In the next section, we first briefly introduce NMA, and we then summarize the main characteristics that have already been described for NMAs published in the medical science field.

## Network meta-analysis

An important underlying idea in NMA is that, besides direct evidence for a comparison between two treatments, the literature may also provide indirect evidence. Direct evidence refers to the effect sizes (e.g., Cohen’s *d*) that can be (directly) calculated from a study that (directly) compares treatment A with a control group, ${d}_{AC}^D$, or that compares two set of treatments, for instance treatment A and treatment B,${d}_{AB}^D$. The superscript stands for “direct,” and the subscript refers to the treatments compared. Indirect effect estimates can be derived by subtracting the direct effects of studies that share a common comparator (Bucher et al., 1997). For instance, if a study compares treatment A with a control group (${d}_{AC}^D$), and another study compares treatment B with a control group (${d}_{BC}^D$), the estimate of the indirect effect of treatment A on treatment B can be calculated by the formula

1 $${d}_{AB}^I={d}_{AC}^D-{d}_{BC}^D$$

However, Eq. 1 is only correct under the transitivity and consistency assumptions. Transitivity implies that, in order to be able to obtain an indirect effect of treatment A on treatment B through a control group C, studies have to be homogeneous in terms of effect modifiers, that is, in terms of participants, interventions, and setting characteristics (Jansen & Naci, 2013). If, for instance, the control group C when compared with treatment A is different from that when compared with treatment B, then the transitivity assumption does not hold. The transitivity assumption is typically checked by qualitatively evaluating the similarity of study characteristics across studies that compare different combinations of treatments (e.g., similar sample, instruments, methods).

The statistical manifestation of transitivity is known as consistency (Cipriani et al., 2013). It is often the case that for a given comparison, both direct (e.g., ${d}_{AB}^D$) and indirect effects (e.g., ${d}_{AB}^I$) are available for the meta-analysis. If the transitivity assumption is met—or in other words, if the characteristics of the studies are similar—we can expect to find that direct and indirect effects are statistically consistent; that is, ${d}_{AB}^D\approx {d}_{AB}^I$. However, if the transitivity assumption does not hold, inconsistency effects (i.e., statistical differences between direct and indirect effects for a given comparison) might emerge (Lu & Ades, 2006). In inconsistent networks, the reliability of indirect effects is low and their interpretability is more difficult.

Once the transitivity assumption is met, the researcher can proceed with the main analyses. In its simpler statistical formulation, a NMA is a meta-regression where indicator variables are introduced to estimate the effect of a given treatment relative to a reference treatment, typically a control group (Salanti, Higgins, et al., 2008). For a comprehensive review of this and other more complex models to carry out NMA, we refer to Efthimiou et al. (2016), Nikolakopoulou et al. (2020), and White et al. (2012).

The methods currently used to detect and quantify inconsistency can be divided into two groups: global and local tests. In general, global tests aim to detect inconsistency within the overall network, whereas local tests aim to identify the specific part of the network where inconsistency exists. The most popular global tests are the design-by-treatment model (Higgins et al., 2012; Jackson et al., 2014, 2016; White et al., 2012), which defines and estimates inconsistency as the difference or variability between treatment effects that stems from different study designs^1^, and the unrelated mean effects model (Dias et al., 2013), which consists in comparing the model fit of a model that assumes consistency with that of a model that allows for inconsistency. The most popular local tests are the loop-specific approach (Bucher et al., 1997; Lumley, 2002), which statistically tests the difference between the direct and indirect effects derived from each closed loop of the network, and the node-splitting (or side-splitting) approach (Dias et al., 2010), which consists in separately synthesizing the direct and indirect effects for a given comparison and statistically comparing the two estimates. For an in-depth explanation of these and other methods for detecting inconsistency, we refer the reader to Efthimiou et al. (2016). When inconsistency effects are detected, the researcher has to find possible explanations, for instance, by searching again for any differences between study characteristics or by detecting problematic studies (e.g., studies at high risk of bias) that lead to inconsistent effects through local methods. If differences between effect modifiers are found across studies, an alternative approach is to include moderator variables in the meta-regression to explain the observed variability that is due to inconsistency (Donegan et al., 2017).

In NMAs, it is also of interest to explore the network geometry through the inspection of the network plot (Salanti, Kavvoura, et al., 2008). In a network plot, all the intervention and control groups are depicted in nodes (whose size is typically proportional to the number of participants that received that intervention/control), and edges connect the nodes that have been directly compared in primary studies (whose width is typically proportional to the number of times those nodes have been directly compared). By visually inspecting the network plot, it is possible to get an idea of the evidence available for each comparison. With all this information, specific recommendations can be given for future studies to fill in gaps in the field (Catalá-López et al., 2014).

In NMA it is also common to rank treatments according to their average effectiveness using procedures such as SUCRA [surface under the cumulative ranking curve] (Salanti et al., 2011) or P-scores (Rücker & Schwarzer, 2015). Also, as in any other meta-analysis, the presence of publication bias has to be checked, and specific approaches have been developed for NMA—for instance, the comparison-adjusted funnel plot (Chaimani & Salanti, 2012). Finally, several statistical programs are available to carry out NMA, including Stata, OpenBUGS, WinBUGS, or R software (Neupane et al., 2014).

## Characteristics of NMAs in the medical science field

Several systematic reviews have described the characteristics of the NMAs published in the medial science field, and their findings are summarized in Table 1. According to Lee (2013), who reviewed 201 NMAs, the term most commonly used to refer to this methodology is “mixed-treatment comparison,” but a more recent review (Zarin et al., 2017) has found that “network meta-analysis” is now the preferred term. Most of the existing systematic reviews published in this field (Bafeta et al., 2013; Lee, 2013; Nikolakopoulou et al., 2014; Petropoulou et al., 2016; Veroniki et al., 2013; Zarin et al., 2017) found similar median numbers of trials included in the NMAs (between 20 and 23), and similar median numbers of treatments compared in NMAs (between 5 and 7). Nikolakopoulou et al. (2014), who examined 186 NMAs, also reported the median sample size per network, which was 7729.

**Table 1:** Characteristics of NMAs published in the medical sciences field and in the psychology and educational sciences domain

	Medical sciences	Psychology & educational sciences
Term to refer to NMA	Network meta-analysis ^g^	Network meta-analysis
Number of trials or studies	Range from 20 to 23 ^a,b,c,d,f,g^	Median: 29
Sample size per network	Median: 7729 ^c^	Median: 2501
Number of treatments and control groups per network	Range from 5 to 7 ^a,b,c,d,f,g^	Median: 8
Geometry of the network	From 19% to 27% were star-shaped networks ^c,d^ Around 85% of the networks included multi-arm studies ^c^	10% were star-shaped networks 90% included multi-arm studies
Multi-arm studies	Number of three-arm studies ranged from 0 to 12 ^f^ Number of four-arm studies ranged from 0 to 6 ^f^	Median number of three-arm studies: 4 (range from 0 to 30) Median number of four-arm studies: 0 (range from 0 to 9).
Effect size most commonly synthesized	Odds ratio ^d^	Standardized mean difference
Transitivity assessed	33% of the studies assessed transitivity, and this percentage increased to 85% from 2015 ^d^	54.77% of the studies assessed transitivity
Inconsistency assessed	23%, 66%, or 51% of the studies checked for inconsistency ^f,c,g^	88.10% of the studies checked for inconsistency
Method to assess inconsistency	Loop-specific approach ^c,d^	Node-splitting approach
Percentage of inconsistent networks	12%, 20% ^e,f^	27.54%
Network meta-analysis model	Bayesian framework ^c, d^	Multivariate meta-regression
Type of model fitted	Random-effects model ^d^	Random-effects model
Method to rank treatments	Probability of being the best ^d^	SUCRA
Method for testing for publication bias	Funnel plots and regression tests ^d^	Comparison-adjusted funnel plots

NMA: network meta-analysis; Superscripts indicate the studies that reported that information: ^a^ Bafeta et al. (2013) ; ^b^ Lee (2013) ; ^c^ Nikolakopoulou et al. (2014) ; ^d^ Petropoulou et al. (2016); Song, Chen, et al. (2011a), ^f^ Veroniki et al. (2013); ^g^Zarin et al. (2017)

As for the geometry of the networks, Nikolakopoulou et al. (2014) found that 19% of the networks were star-shaped; that is, primary studies included in these meta-analyses were two-armed and always compared a treatment group against a control or placebo, but never compared treatments directly. A higher percentage of star-shaped networks was reported by Petropoulou et al. (2016): out of the 456 NMAs reviewed, 27% were star-shaped networks, although the authors observed a marked decline in the publication of this type of network analysis from 2005 to 2015. Regarding non-star-shaped networks, Nikolakopoulou et al. (2014) noted that 85% of them included at least one multi-arm study. Veroniki et al. (2013), who evaluated 40 NMAs of binary outcomes, reported that the number of three-arm trials included per network ranged from 0 to 12, whereas the number of four-arm trials ranged from 0 to 6.

Regarding the effect size most commonly synthesized, Petropoulou et al. (2016) found that the majority of the NMAs used binary outcomes, specifically odds ratio (39% of the 456 NMAs reviewed), and that 20% used mean differences. Similarly, Nikolakopoulou et al. (2014) found that the majority of the NMAs used dichotomous outcomes (60%) such as odds ratios, and that only 28% of the NMAs used a continuous outcome, most often mean differences, followed by standardized mean differences and ratio of the means.

Moving on to how these NMAs have addressed the main assumptions of network meta-analysis, namely transitivity and consistency, Petropoulou et al. (2016) found that 77% of the NMAs did not mention anything related to the transitivity assumption, and that 28% of the NMAs did not report the use of any method to assess inconsistency. However, if these results were disaggregated by year of publication, a substantial improvement was observed: from the NMAs published in 2015, 85% did discuss transitivity and consistency, and 74% used adequate methods to statistically address inconsistency. Veroniki et al. (2013) found similar results, where only 23% of the researchers were aware of the consistency tests and applied them, and Nikolakopoulou et al. (2014) also reported that 44% of the reviewed studies did not test for inconsistency. More optimistic was the information provided by Zarin et al. (2017), which showed that 53% of the 456 NMAs reviewed discussed and/or assessed consistency in some way, and 51% explicitly addressed inconsistency or an unequal distribution of treatment effect modifiers across different comparisons.

As for the methods most commonly used to evaluate inconsistency, Nikolakopoulou et al. (2014) reported that 32% of the NMAs did evaluate consistency through the loop-specific approach (14%), 7% used the Lumley model (i.e., linear model with random effects for specific comparisons), 2% applied the node-splitting method, 2% used the unrelated mean effects model, and finally only 1% used the Lu and Ades model (i.e., a NMA model that includes one inconsistency factor for each loop). The loop-specific approach was also the preferred approach in the NMAs reviewed by Petropoulou et al. (2016), followed by the node-splitting approach. In this review, the design-by-treatment approach was applied in only a few NMAs. It is worth mentioning that 24% and 27% of the NMAs reviewed by Nikolakopoulou et al. (2014) and Petropoulou et al. (2016), respectively, used inadequate methods to assess inconsistency (e.g., comparing NMA estimates with direct estimates or with estimates published in previous meta-analyses).

Nikolakopoulou et al. (2014) found that combining direct and indirect effects was done primarily from a Bayesian framework (59%), followed by a meta-regression approach (15%) and the adjusted indirect comparison method proposed by Bucher et al. (1997, 15%). However, these authors complained about the small numbers of articles that specified the method used to carry out the NMA. Petropoulou et al. (2016) also found that Bayesian hierarchical models were the preferred method (64%), but the second most common approach was the Bucher method (18.8%), followed by meta-regression (9.4%). More complex models, such a multivariate meta-regression, were applied to only a very small extent according to this systematic review. This review also reported that 56% of the NMAs tried to disentangle heterogeneity through subgroup analysis, meta-regression, and/or sensitivity analyses.

Regarding the type of model fitted (fixed- versus random-effects model), Petropoulou et al. (2016) noted that 50% of the NMAs fitted a random-effects model and 37% fitted a fixed-effects model*.* From the NMAs where a random-effects model was fitted, only 13% explained whether a common between-studies variance was assumed for all treatment comparisons.

According to the review by Petropoulou et al. (2016), 43% of the studies ranked treatments, where the “probability of being the best” was the preferred method, followed by SUCRA. Lastly, regarding publication bias analyses, Petropoulou et al. (2016) report that 31% of the studies examined the existence of small-study effects through funnel plots or regression tests, and that only a few NMAs applied extensions of these approaches to the NMA context.

## Method

### Search procedure

Databases were searched in March 2021 for NMAs published in the field of psychology and education. For the initial search, five electronic datasets were used: Web of Science^2^, ProQuest Psychology Database, Scopus, Science Direct, and ERIC (Education Resources Information Center). The following search string was used: “(network OR mixed treatment* OR multiple treatment* OR mixed comparison* OR indirect comparison* OR simultaneous comparison*) AND (meta-analysis)”^3^. The search was limited to the psychological and educational sciences field by selecting specific field filters in each database. The strings used and the filters applied on each dataset can be found in the supplementary material (www.osf.io/w82kj). Also, we asked research groups that we knew were conducting a NMA for full manuscripts that were submitted for publication but not yet published. To check for a possible language bias, an additional, abbreviated search was done in all databases using the term “network meta-analysis” translated into five different languages: Spanish, German, Dutch, French, and Chinese. More details about this abbreviated search can be found in the supplementary material.

### Inclusion and exclusion criteria

The search was not restricted to any time interval. A NMA was included if it met the following criteria: (a) Two or more interventions were compared; component network meta-analyses and NMAs on individual participant data were excluded. (b) It was reported as a journal article (or it was submitted for publication), conference paper or dissertation; posters, books, or any other format were excluded. (c) The research area of the NMA was within the field of psychology and education, meaning that the dependent variable was a psychological or educational outcome, and/or the interventions applied were educational or psychological; NMAs published in other domains such as medical sciences or life sciences were excluded, as were studies in which the outcome was psychological (e.g., a given disorder) but the interventions or treatments were only pharmacological. Some NMAs included a mix of pharmacological and psychological interventions, and they were only included if at least 20% of the treatments were psychological or educational interventions. (d) The article was written in Spanish or English.

### Data extraction

The information extracted from each study was coded in a spreadsheet, where four blocks could be differentiated. The first block consists of basic information regarding the paper: author’s name, title, date of publication, and journal. The second block includes the main characteristics of the meta-analytic dataset: number of studies and number of effect sizes synthesized, total sample size, number of treatments compared and number of control groups, total number of multi-arm studies, type of effect size, and the type of geometry of the network. The total number of effect sizes analyzed in each NMA included all the possible effect sizes that can be derived from a study.

**Fig. 1:**
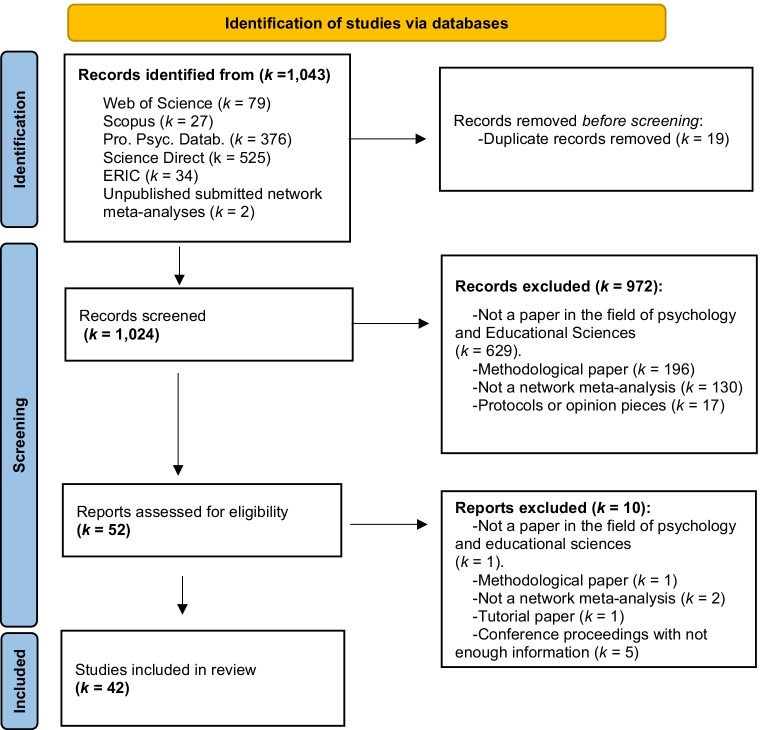
PRISMA flow diagram of the study selection process

That is, if a study had two intervention groups and a control group, then three effect sizes were accounted for in that study, although for most software it suffices to include just two effect sizes because the third can be easily derived. The geometry of the network was described using the indicators proposed by Salanti, Kavvoura, et al. (2008), namely (limited) diversity and (significant or nonsignificant) co-occurrence. These indicators are calculated using the number of treatments and the number of comparisons available for each pair of treatments. However, we were unable to calculate them because the number of comparisons per pair of treatments was hardly ever available. Therefore, we visually assessed these two dimensions by examining the network plot based on the examples provided by Salanti, Kavvoura, et al. (2008, see Fig. 1). Specifically, networks with more than seven nodes were considered diverse, and if two interventions (or one intervention and a control group) were more often compared in the literature than any other pair of treatments (i.e., the line connecting two nodes in the network plot was substantially thicker than the other lines), then we concluded that there was co-occurrence. Also, we coded whether the network was well connected or disconnected. The network was “well connected” when at least 50% of the interventions were compared head-to-head. A third block gathered information on how assumptions were handled and which methods were used to carry out the NMA: whether and how the transitivity assumption and consistency were addressed, how indirect evidence was derived, which strategies were used to deal with inconsistent effects, whether the method was applied from a frequentist or a Bayesian approach, the type of model fitted (fixed- or random-effects model), whether—if a random-effects model was selected—a common between-studies variance was assumed for all comparisons, whether treatments were ranked and which method was used, whether moderator analyses were performed within the network analyses, whether publication bias was assessed, what software was used, and finally which terminology was used to describe the type of analysis. Finally, in a fourth block we coded whether researchers complied with two of the main principles of open science (i.e., reproducibility and transparency) by providing access to the full dataset and to the code used to analyze it.

Some papers reported several NMAs, typically because authors analyzed several outcomes (e.g., effectiveness of an intervention and acceptability) or because the same outcome was measured at different time points (e.g., effectiveness immediately after treatment and after 6 months). For this systematic review, only the characteristics of the NMAs of the main outcomes were coded, and only measures immediately after the treatment. The complete dataset can be found at www.osf.io/w82kj

## Results

A total of 1041 entries were identified across the five electronic datasets. After screening of titles and abstracts, 991 studies were removed. Of those 991 entries, 130 were not NMAs, 196 were methodological papers, 629 were NMAs within a field other than psychology and educational sciences, 17 were protocols or opinion pieces, and 19 were duplicates. Therefore, 50 entries were further screened by reading the full text. From this second screening, ten studies were removed because they were conference proceedings without sufficient information^4^ (*k =* 5), tutorial papers (*k =* 1), not NMAs (*k =* 2), methodological papers (*k =* 1), or not within the field of psychology and educational sciences (*k =* 1). Therefore, after the second screening a total of 40 studies were selected for further coding. Finally, two unpublished NMAs (submitted for publication) were added, leading to a total of 42 NMAs. No study was retrieved from the search done using the translation of “network meta-analysis” into five different languages (more information can be found in the supplementary material, in the search procedure .pdf file). The Preferred Reporting Items for Systematic Reviews and Meta-Analyses (PRISMA) flowchart in Fig. 1 summarizes the study selection process (Moher et al., 2009). A list of the excluded studies (and the reason that they were excluded) can be found in the supplementary material: www.osf.io/w82kj

### Main characteristics of the meta-analytic datasets

**Fig. 2:**
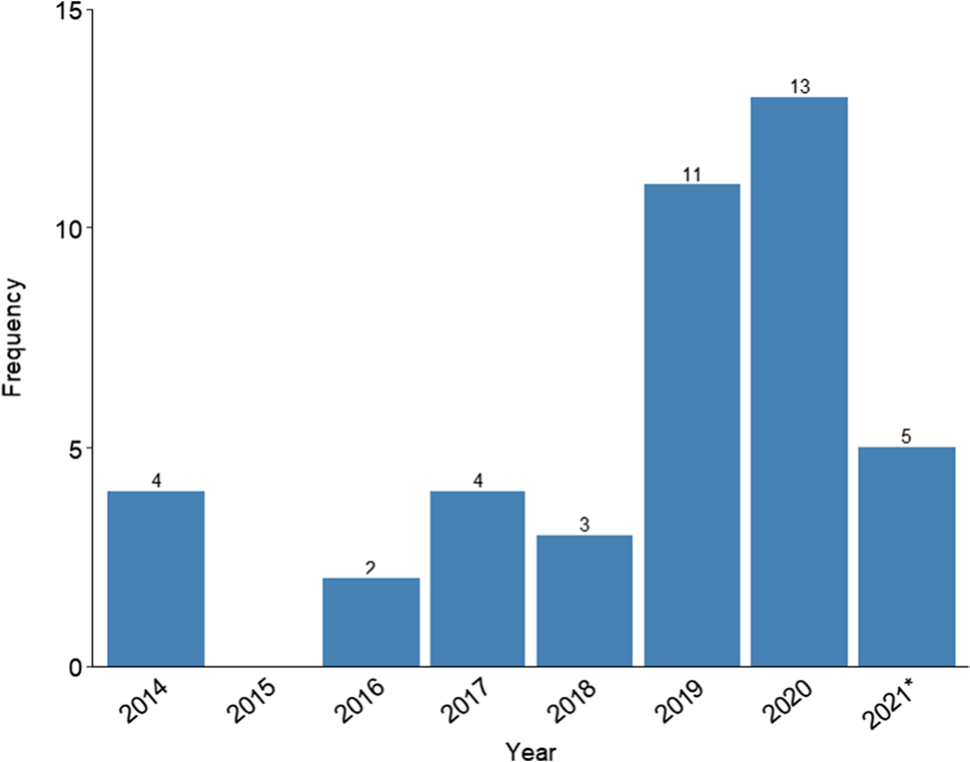
Number of NMA studies published each year. *Note*. The search was done in March 2021, so the bar for 2021* only includes data from January to March 2021

The main characteristics of NMAs published in the field of psychology and education can be found in Table 1. The first study with the term “network meta-analysis” in the title in the field of psychology and education was published in 2014. Figure 2 shows a noticeable increase in the number of publications since 2019. All but one study used the term “network meta-analysis” to refer to this methodology, with only one using the term “multiple treatments meta-analysis.” The vast majority of the studies were from the field of clinical psychology (*k =* 34), and the rest were from the fields of educational science (*k =* 2), social psychology (*k =* 2), clinical and cognitive psychology (*k =* 2), and cognitive psychology (*k =* 1). These 42 studies included a total of 80 NMAs^5^. Most studies carried out a NMA of only one primary outcome (*k* = 24), ten studies included the NMAs of two different outcomes, and eight studies included three or more NMAs (with a maximum of nine different NMAs).

Table 2 contains descriptive statistics, i.e., the number of primary studies included in each NMA, the total number of effect sizes included in each NMA, the number of primary studies with three-arm trials and/or four-arm trials or more, the number of interventions and control groups included in each network, the total number of nodes (interventions + control groups), and the total sample size. On average, a prototypical NMA in the psychological and educational sciences field would include 29 primary studies, with a total of 30 effect sizes and 2501 participants. From these 29 studies, four might include more than one intervention (i.e., three-arm trial). Typically, the network included eight nodes: six intervention and two control groups. It should be noted that the distribution of the number of studies and the total sample size is positively skewed, meaning that there are some NMAs with a larger number of trials (third quartile = 65.50) and larger number of participants (third quartile = 6285). From the NMAs that reported the total number of multi-arm studies (*l =* 56), only four (7.14%) included only two-arm studies.

**Table 2:** Descriptive statistics of the main characteristics of the datasets analyzed in each of the 80 NMAs

	Min.	1^st^ Q	Median	Mean	3^rd^ Q	Max.	Missing
Number of trials	5	16	29	45.29	65.50	291	5
Number of effect sizes	8	23	39	65.68	86	351	11
Number with three arms	0	2	4	6.45	9	30	24
Number with four or more arms	0	0	0	1.20	1.25	9	24
Number of intervention groups	1	5	6	7.82	10	28	1
Number of control groups	1	1	2	2.44	3	7	1
Number of nodes	3	6	8	10.05	13	32	1
Total sample size	420	1194	2501	4177	6285	15,191	14

Min. = minimum; 1^st^ Q = first quartile; 3^rd^ Q = third quartile; Max. = maximum

Based on the data from Table 2, it is possible to make a rough estimate of the average number of studies that reported a specific treatment comparison. The median number of nodes studied in a NMAs is eight, meaning that primary studies within a NMA could potentially report information in one or more comparisons out of the 36 possible comparisons [(8×9)/2]. The number of effect sizes included in a NMA is on average around 66, meaning that on average there are two effect sizes giving information about each treatment comparison. However, it should be kept in mind that this would only apply to geometric networks where co-occurrence does not occur, which as we will see in the following paragraph is not often the case.

Moving on to the network geometry, the network graph was available in 70 NMAs (10 NMAs did not report any network plot). Seven of these NMAs were star-shaped (10%). From the 63 networks that had closed loops, most of them were diverse and co-occurrence occurred (*l* = 39); that is, there were more than seven nodes, and two of them were more connected (i.e., thicker line) than the other nodes. In 14 NMAs there were fewer than seven nodes (limited diversity) and co-occurrence occurred; in six NMAs there was limited diversity (fewer than seven nodes) and no co-occurrence occurred. Finally, in four NMAs, there was diversity and no co-occurrence. Regarding the connectivity of the network, 80.95% of networks were well connected (*l* = 51), and in 19.05% of the networks (*l* = 12) the connectivity was poor.

In 61 networks (76.25%) the scale of the outcome was continuous, while in 19 networks (23.75%) the outcome measured was dichotomous. The effect sizes used to quantify the magnitude of the difference between two means were standardized mean difference (*l =* 37), standardized mean change (*l* = 21), and unstandardized mean difference (*l* = 2). Also, there was one NMA that used Pearson correlation as the unit of analysis. For the 19 NMAs in which the main outcome was dichotomous, the most common effect size was the odds ratio (*l* = 12), followed by the relative risk (*l* = 7).

### Methodological characteristics of the network meta-analyses

The transitivity assumption was not addressed in 45.23% of the studies (*k* = 19). From the 23 studies that explicitly addressed the transitivity assumption, four did it incorrectly by assessing transitivity through the evaluation of inconsistency; that is, the authors considered transitivity and consistency as the same assumption. From the remaining 19 studies that handled transitivity using adequate methods, the main strategies were as follows: (1) exploring whether effect modifiers were similarly distributed across studies (*k* = 9); (2) imposing very restrictive inclusion criteria regarding the characteristics of the sample and the intervention so that studies were homogeneous (*k* = 3); (3) creating several subgroups of studies with homogeneous characteristics (*k* = 3); (4) selecting only a subgroup of homogeneous studies and excluding the rest of the studies from the analysis (*k =* 2); (5) combining strategies 2 and 4, i.e., first imposing very restrictive inclusion criteria and then removing those studies that were heterogeneous (*k* = 2).

Although only 54.77% of the studies addressed the transitivity assumption, most studies did assess the presence of inconsistency between direct and indirect evidence (*k* = 37, 88.10%). In three studies, inconsistency could not be assessed because there was only indirect evidence available for intervention effects (i.e., star-shaped networks), and only two studies did not address the inconsistency assumption. From the 37 studies that explored the presence of inconsistency, seven used up to three methods to detect and quantify inconsistency, 12 studies used two methods, and 18 studies used only one method to detect inconsistency. The most commonly used method was the node-splitting approach (*k* = 19), followed by the strategy of comparing the model fit of a model assuming consistency and a model assuming inconsistency (*k* = 14), the design-by treatment method (*k* = 10), and the loop-specific approach (*k* = 10). Other strategies that were used to a lesser extent included quantifying the total heterogeneity through the use of the well-known Q statistic (Cochran, 1954; *k =* 1), the *H*^2^ statistic (*k* = 1), or the *I*^2^ statistic (Higgins & Thompson, 2002; *k* = 1); applying the decomposition of the heterogeneity statistic (Krahn et al., 2014); using visual tool plots as the net-heat plots (Krahn et al., 2013; *k* = 1) or extended forest plots that show the results of the network meta-analysis for each comparison (see for instance Riley et al., 2017; *k* = 1); comparing the amount of heterogeneity between the consistency and inconsistency model instead of comparing the goodness of fit (*k* = 1); and finally, in one meta-analysis, due to the small number of studies in the sample, by qualitatively checking the homogeneity of data (*k =* 1).

Of the *l =* 69 networks where inconsistency was evaluated (in 11 networks, either consistency was not assessed or the network was star-shaped), in 50 networks (72.46%) the authors did not find statistical indicators of inconsistency, in 17 network (24.32%) statistical inconsistency was found, and in two networks (from the same study) results were inconclusive because the authors gave the numerical results of the inconsistency analyses but did not interpret them. When inconsistency effects were found, the authors handled them using the following strategies: (1) dividing primary studies into homogeneous groups based on characteristics of the interventions and/or participants and/or settings, and carrying out different NMAs on each of these subsets (*k* = 3); (2) mentioning in the discussion the limited interpretability of the NMAs due to the existence of inconsistent effects (*k* = 3); (3) including effect modifiers within the meta-regression to control for differences in intervention, participants, or setting characteristics across studies (*k* = 3); (4) checking for coding errors and reviewing those studies that “provoked” inconsistency, and either removing the study or creating an extra node for that study (*k =* 2). Finally, two NMAs did not report the strategy implemented to deal with inconsistent effects.

Regarding the NMA techniques, in 31 NMAs the authors carried out a multivariate meta-regression that took into account the presence of multi-arm studies, and in 11 NMAs this information was not reported. Half of the NMAs used a frequentist approach (*k* = 21, 50%), and among these NMAs the preferred software was Stata (*k* = 10), closely followed by R software (*k =* 9). One study used both R software and Stata, and the R package most commonly used was *netmeta* (*k* = 9, Rücker et al., 2020), and to a lesser extent *metafor* (*k* = 1, Viechtbauer, 2010). Finally, one study used a macro in Excel called MetaXL. A Bayesian approach was implemented in 19 NMAs, and one study used both frequentist and Bayesian approaches. When a Bayesian approach was used, the authors commonly used more than one kind of software (that is why the following numbers sum to more than 19). The software most used was WinBUGS (*k =* 9), followed by OpenBUGS (*k* = 7), R software (*k* = 6) with the *getmc* package (Van Valkenhoef & Kuiper, 2021), and JAGS (*k* = 3).

Most NMAs fitted a random-effects model (*k* = 37), only one NMA fitted a fixed-effects model (because no relevant heterogeneity was observed), one NMA reported results from both fixed- and random-effects models, and another NMA used an inverse-variance heterogeneity model (Doi et al., 2015). Two NMAs did not report information on the type of model fitted. From those NMAs in which a random-effects model was fitted, 54.05% (*k* = 20) explicitly stated that a common between-studies variance was estimated across treatment comparisons, whereas this information was not available for the remaining random-effects NMAs (*k =* 17, 45.95%).

In many studies, the authors applied techniques to rank the treatments from the best to the worst. The most popular technique was SUCRA, which was implemented in 55.38% of the NMAs (*k* = 22). Another 30.95% (*k* = 13) of the NMAs did not rank the treatments, 11.90 % (*k* = 5) calculated P-scores, and two studies (4.76%) did not report the method they used to derive the ranking of the treatments.

Moving on to the examination of the possible presence of publication bias, 22 NMAs (52.28%) did not apply any technique to check whether publication bias could exist. In 14 NMAs (33.33%), the authors applied one method to detect publication bias, with the most popular being visual inspection of comparison-adjusted funnel plots (*k* = 8), followed by the Egger regression test (*k* = 3). In 15 NMAs, the authors applied two methods to detect publication bias, the most popular being the combination of the visual inspection of funnel plots for each comparison and the Egger regression test (*k* = 7). Five NMAs combined the visual inspection of comparison-adjusted funnel plots with the application of the Egger regression test. In one NMA, the Egger regression test was combined with the visual inspection of contour-enhanced funnel plots, another NMA combined comparison-adjusted funnel plots with Begg’s rank correlation test, and finally another NMA used visual inspection of comparison-adjusted funnel plots and several standard funnel plots (one for each comparison).

Moderator or subgroup analyses were carried out within the network analyses in 14 studies (33.33%). In two NMAs it was unclear how many moderators were tested within the multivariate meta-regression, and in one NMA subgroup analyses were performed instead of moderator analyses because the software did not allow for moderators. In the remaining 11 NMAs, a median of five moderator variables were tested, with a minimum of one and a maximum of ten. In only two NMAs, the authors explained that moderator variables were introduced as interaction terms in the multivariate meta-regression, and no study indicated whether moderator variables were introduced at the same time in the regression model or one by one.

Finally, regarding the analysis of open science research practices, we found that more than half of the studies did not provide the dataset (*k =* 24, 57.14%), 16 studies (38.10%) did provide access to the full dataset, and in two NMAs the authors indicated that the dataset was available upon request. As for the availability of the code used to carry out the NMA, 38.10% of the studies (*k* = 16) did provide the R, OpenBUGS, or WinBUGS code used, but most NMAs did not report it (*k* = 26, 61.90%).

### Comparison of characteristics of NMAs across fields

Table 1 summarizes the characteristics of NMAs published in the medical science field described in previous systematic reviews, and the characteristics found in this study of NMAs published in the psychological and educational sciences domain.

Starting with the characteristics of the meta-analytic datasets, we have found that the median number of studies included in NMAs published in the field of psychology and education seems to be larger than that observed for NMAs in the medical sciences domain. However, the median sample size of a NMA from the field of psychological and educational sciences is smaller (2501) than the one reported by Nikolakopoulou et al. (2014) for the medical sciences (7729). Therefore, although more studies are typically available for NMAs in psychology and education, the sample size of these studies seems to be smaller. Also, the number of “nodes” (intervention + control groups) is slightly larger in the psychology and educational sciences field. Regarding the geometry of the networks, we have found a smaller percentage of star-shaped networks in NMAs from the field of psychology and education. For those NMAs that include studies that compare multiple interventions, the range of multi-arm studies in NMAs from psychology and educational sciences is wider than that found for NMA from the medical science field. Finally, the effect size most commonly used in the NMAs reviewed in this study is the standardized mean difference, whereas in NMAs from the medical science field, the effect size most commonly used is the odds ratio.

Moving on to the methodological characteristics, we have found that only half of the NMAs explicitly address the transitivity assumption, whereas in NMAs in the medical science field, this assumption is addressed in most of the NMAs published from 2015. In contrast, the majority of the studies in psychological and educational sciences field do test for the presence of inconsistent effects, whereas only half or less of the NMAs in the medical science field carried out methods to assess inconsistency between direct and indirect evidence.

Regarding the methods used to test for inconsistent effects, in NMAs from the medical science field, local tests are more often used (e.g., loop-specific approach), whereas in NMAs from the psychological and educational sciences field, after the node-splitting approach, the second and third most popular methods are global methods (i.e., unrelated mean effects model and design-by-treatment approach). Results from these consistency tests revealed that around a quarter of the networks published in psychology and educational sciences are inconsistent. However, in the medical sciences field, the percentage of inconsistent networks is smaller (see Table 1).

Half of the NMAs in psychology and educational sciences use a frequentist approach, fitting a multivariate meta-regression, whereas in the medical sciences, researchers more often opt for fitting a hierarchical random-effects model within a Bayesian framework. In both fields, it is common to fit a random-effects model instead of a fixed-effects model. However, these fields differ in the methods used to rank the treatments and to detect publication bias: whereas in the medical science field authors mainly use “probability of being the best” as a ranking technique and standard funnel plots to detect publication bias, NMAs from the field of psychology and educational sciences mainly use the SUCRA technique to sort treatments by their average effectiveness and comparison-adjusted funnel plots to test the presence of publication bias.

## Discussion

The goal of the present manuscript was to describe the main characteristics of the NMAs published in the field of psychology and education, and to determine whether these characteristics differ from those observed in NMAs in the medical science.

As described in the previous sections, several differences are found between fields: NMAs from the psychology and educational sciences domain include, on average, more studies with smaller sample sizes. Also, more treatments and control groups are compared, so one is less likely to observe star-shape networks (in other words, networks where none of the interventions are directly compared). It seems that in the fields of psychology and education, competing interventions are directly compared more often, maybe because psychological therapies are easier to implement than some pharmacological or medical therapies. Most of the networks were found to be well connected, which is positive in terms of the reliability of the estimates provided by these networks (Mills et al., 2013). However, a deeper analysis of the geometry of the networks shows that co-occurrence happens quite often; that is, it is common to find that several primary studies report direct evidence on the comparison of two nodes, whereas other (direct) treatment comparisons are based on only a few or even just one study. On one hand, this finding could indicate that publication bias exists (e.g., no differences are found among some interventions and therefore those studies never get to be published), or that authors tend to have a preference for comparing certain interventions with others. On the other hand, co-occurrence could also occur because some interventions are more novel (and therefore there are fewer primary studies implementing them) or are easier to implement (Salanti, Kavvoura, et al., 2008). Even so, it is important that future simulation studies take into account this very common scenario in which the distribution of studies across comparisons is quite imbalanced, because it directly impacts the amount of inconsistency observed (Veroniki et al., 2014) while reducing the statistical power to detect inconsistency (Mills et al., 2011). A final comment regarding network geometry is that none of the studies included in this review reported the numerical indicators provided by Salanti, Kavvoura, et al. (2008) to describe the network geometry. We recommend that researchers calculate these indexes in future NMAs in order to have objective measures of the geometry of the network.

A remarkable finding is that most NMAs in the field of psychology and education did not check whether the transitivity assumption was met, but they did apply methods to check whether effects were inconsistent. Interestingly, this trend was reversed in NMAs in the medical sciences field: in most of the NMAs, the transitivity assumption was checked, but the consistency assumption was not tested (Nikolakopoulou et al., 2014; Veroniki et al., 2013; Zarin et al., 2017). The fact that most studies in psychology and educational sciences did not check the transitivity assumption is especially worrying, because the reliability of the NMA results directly depends on this assumption (Salanti, 2012). To explore whether the transitivity assumption holds, researchers have to check whether the characteristics of the studies are similar enough so that the reference group (which is often the control group) can be considered equivalent across studies. These characteristics encompass all the features of the studies that might affect the results observed: characteristics of the sample (e.g., percentage of men/women, average age, nationality, culture), the design (e.g., longitudinal or transverse study), the instruments (e.g., standardized tests, self-report measures), the data collection (e.g., random or convenience sampling), and so on. A good example of a NMA that did properly address the transitivity assumption is the one published by Cuijpers et al. (2019), who created a table that summarized important study characteristics for each possible comparison in the network (see their eAppendix H).

Another positive finding is that most researchers in the psychological and educational sciences field did apply consistency tests. Indeed, they did it to a greater extent than researchers in the medical science field. However, it should be kept in mind that researchers in the field of medicine started applying NMA in the early 2000s, when the number of resources available (e.g., tutorial papers, software, and bibliography on the topic) was much smaller. In this regard, it is understandable that methodological practices were slightly worse in the medical science field, and at the same time it is less justifiable that many NMAs in psychology and educational sciences still do not comply with the main assumptions.

Among the NMAs explored in this study, half used more than one method to test for inconsistency effects, often combining a global method (e.g., unrelated mean effects model) with a local method (e.g., node-splitting approach). Applying different methods to detect inconsistent effects is an advisable practice given their low power (Salanti, 2012; Veroniki et al., 2014). In this context, our recommendation is to apply both a global test to first determine whether the network is inconsistent, and then a local test to identify the particular comparison where inconsistency occurs*.* Some of the NMAs (Cuijpers et al., 2019; Fodor et al., 2020; Mavranezouli et al., 2020a, b) reviewed in this study did carry out both local and global tests, so we refer the reader to these studies as a reference of how to apply and interpret both local and global inconsistency tests. Finally, it is worth noting that, according to this review, inconsistent networks might be more prevalent in psychology and education than in the medical field, thus reinforcing the importance of applying tests that detect these inconsistencies between direct and indirect effects.

Another difference observed across fields is the approach used to carry out the analysis: in psychology and educational sciences it is more common to use a frequentist approach (i.e., *netmeta* in R), whereas in the medical sciences it is more common to use a Bayesian approach (Nikolakopoulou et al., 2014). Only small differences are expected between the parameter estimates yielded by the frequentist and Bayesian approaches (Efthimiou et al., 2016). Even so, Bayesian approaches are more flexible regarding the incorporation of moderator variables in the statistical model, although the *metafor* package in R (Viechtbauer, 2010) also allows the inclusion of moderator variables from a frequentist approach (however, none of the NMAs reviewed in this study used this option). It is also worrisome that in many NMAs from both fields, information regarding the specific method used to derive indirect effects was missing (Hutton et al., 2014), thus hampering the reproducibility and replicability of the NMA. Future NMAs in all fields should focus on improving the reporting of the methods used to conduct the NMA. In that regard, the use of the PRISMA statement and its extension to NMA (Hutton et al., 2015) is a key recommendation to improve the quality of reporting.

Following up on the reporting quality issue, in most of the NMAs that performed moderator analyses, the authors did not explain how these moderators were handled within the statistical model. Only two studies explicitly stated that moderator variables were introduced as interactors with the indicator variables in the meta-regression, but for the other 12, it is unknown whether moderators were entered as main or as interaction effects, or whether all moderators were entered at once or one by one. For future NMAs, it is important to keep in mind that when moderators are introduced as interaction effects, it should be first tested and confirmed that direct and indirect effects are consistent for each level of the moderator (see Donegan et al., 2017). Another underreported aspect found in this review is that only half of the NMAs indicated whether a common between-studies variance was assumed for all treatment comparisons. Future NMAs should be more transparent on these more technical issues in order to make their research more transparent and reproducible. Regarding other reproducibility and transparency research-related practices, this systematic review found that less than half of the NMAs made their datasets and codes available, preventing other researchers from being able to reanalyze or reproduce the results, and therefore hampering the credibility of their scientific findings (Vazire, 2017). Although this rate of data- and code-sharing is much higher than that reported in other reviews (e.g., Hardwicke et al., 2020), there is still much room for improvement. Guidelines on how to move to more transparent and reproducible research can be found elsewhere (e.g., Klein et al., 2018; Lowndes et al., 2017; Stodden, 2015).

We would like to end the discussion by expressing our concern about the fact that only 50% of the NMAs explored the presence of publication bias. Publication bias is one of the greatest threats to the validity of any meta-analysis, and if no efforts are made to detect it, the conclusions of the meta-analysis might be inflated and therefore no longer reliable. In NMA, the detection of publication bias is more complicated than in a standard meta-analysis, but simple approaches do exist for modeling small-study effects, such as the comparison-adjusted funnel plot (Chaimani & Salanti, 2012), that can be easily implemented in R with the *netmeta* package and in WinBUGS (the code is available in the authors’ manuscript). Finally, it is also of some concern that the vast majority of the NMAs only used keywords in English to search for studies, so only studies written in that language could be retrieved. We encourage future researchers to carry out systematic searches using keywords translated to other languages to avoid a monolingual bias.

The present systematic review is not without limitations. First, we have only included studies that specifically mention in their title the string “network meta-analysis,” “mixed-treatment comparison meta-analysis,” “multiple treatment meta-analysis,” “indirect comparison meta-analysis,” or “simultaneous comparison meta-analysis.” There may be studies that did not indicate in their titles that it was a NMA, and therefore those studies could not be retrieved for this review. Also, most of the NMAs found in this review belong to the clinical psychology domain. Therefore, the results described here may not adequately represent NMAs in other subdomains of the psychology and educational sciences field. In this regard, we hope that more NMAs are published soon in other areas, such as in social or educational psychology, as the comparison of multiple (social or educational) interventions is of interest in these fields as well. Another limitation of the present review is the way “connectivity of the network” was assessed. There are mathematical approaches to “measure” network connectivity (Thom et al., 2019), but raw data are needed to compute those indexes. The approach implemented in this study (i.e., checking whether at least 50% of the interventions had a direct head-to-head comparison with another intervention) is novel, and we cannot ensure that this is a reliable measure of network connectivity.

In conclusion, the characteristics of NMAs in the field of psychology and education seem to differ slightly from those in medicine. Future simulation studies in NMA should take these characteristics into account to make the results more informative to this field. Finally, this review has shown that the methodological aspects of the reviewed NMAs can still be largely improved. In that regard, we encourage researchers to always follow the PRISMA statement extended to NMA (Hutton et al., 2015), and to consult the many tutorials that are already available to carry out a NMA (e.g., Harrer et al., 2019; Mavridis et al., 2015; Molloy et al., 2018; Salanti, 2012; Salanti et al., 2011).

## Supplementary Information

Below is the link to the electronic supplementary material.

Supplementary file1 (XLSX 127 KB)

Supplementary file1 (XLSX 149 KB)

Supplementary file1 (PDF 227 KB)

## Data Availability

The dataset and supplementary materials are available at www.osf.io/w82kj

## Footnotes

1 In NMA, the term “design” refers to the set of treatments that are compared in a given study. For instance, if a study compared treatment A and treatment B, the design would be AB, and if another study compared treatments A, B, and C, then the design would be ABC.

2 The search in Web of Science includes the core collection and the following databases: BIOSIS Citation Index, Current Contents Connect, Chinese Science Citation database, Data Citation Index, Derwent Innovations Index, KCI-Korean Journal Database, MEDLINE, Russian Science Citation Index.

3 This string is based on the search string used in Nikolakopoulou et al. (2014) and Veroniki et al. (2013).

4 First authors were contacted to request full text. Four of them did not reply, and one of them replied saying that the full text was not yet available.

5 Throughout the manuscript, *k* refers to the number of studies analyzed (*k* = 42) and *l* refers to the total number of NMAs included in the 42 studies (*l* = 80)

## Code availability

The code to analyze the dataset is not available.
