# Supplementary material for: Network meta-analysis in psychology and educational sciences: A systematic review of their characteristics
Source: Behav Res Methods. 2022 Jul 11;55(4):2093–108. doi: 10.3758/s13428-022-01905-5 (PMC10250269; doi:10.3758/s13428-022-01905-5)
Supplement: Supplementary file 3 — Supplementary file1 (PDF 227 KB) [file 13428_2022_1905_MOESM3_ESM.pdf]

This document includes more details of the systematic search procedure (**1. Search procedure**) and of the abbreviated search using terms in other languages and its results (**2. Abbreviated search**).

1. Search procedure

Web of Science

Three filters were created and then combined.

1. (TI=network OR mixed treatment* OR multiple treatment* OR mixed comparison* OR indirect comparison* OR simultaneous comparison*)) *AND* IDIOMA: (English OR Spanish)
2. (TI=meta-analys*) *AND* IDIOMA: (English OR Spanish)
3. (SU = (Psychology OR Education & Educational Research)) *AND* IDIOMA (English OR Spanish)

<u>Screenshot of the search:</u>

# 4	79	#3 AND #2 AND #1<br>*Índices=SCI-EXPANDED, SSCI, A&HCI, CPCI-S, CPCI-SSH, ESCI Período de tiempo=Todos los años*
# 3	2.816.513	(SU = (Psychology OR Education & Educational Research)) *AND* **IDIOMA:** (English OR Spanish)<br>*Índices=SCI-EXPANDED, SSCI, A&HCI, CPCI-S, CPCI-SSH, ESCI Período de tiempo=Todos los años*
# 2	150.442	(TI = meta-analys*) *AND* **IDIOMA:** (English OR Spanish)<br>*Índices=SCI-EXPANDED, SSCI, A&HCI, CPCI-S, CPCI-SSH, ESCI Período de tiempo=Todos los años*
# 1	885.809	(TI= (network OR mixed treatment* OR multiple treatment* OR mixed comparison* OR indirect comparison* OR simultaneous comparison*)) *AND* **IDIOMA:** (English OR Spanish)<br>*Índices=SCI-EXPANDED, SSCI, A&HCI, CPCI-S, CPCI-SSH, ESCI Período de tiempo=Todos los años*

Scopus

TITLE(network OR mixed AND treatment* OR multiple AND treatment* OR mixed AND comparison* OR indirect AND comparison* OR simultaneous AND comparison*) AND meta-analys* AND SUBJAREA (arts OR busi OR deci OR econ OR psyc OR soci OR heal OR neur)

<u>Screenshot of the search:</u>

TITLE (network OR mixed AND treatment* OR multiple AND treatment* OR mixed AND comparison* OR indirect AND comparison* OR simultaneous AND comparison*) AND meta-analys* AND SUBJAREA (arts OR busi OR deci OR econ OR psyc OR soci OR heal OR neur)

ProQuest Psychology dataset

TI(network OR mixed treatment* OR multiple treatment* OR mixed comparison* OR indirect comparison* OR simultaneous comparison*) *AND* TI(meta-analys*)

Additional filters: Language English and Spanish

<u>Screenshot of the search:</u>

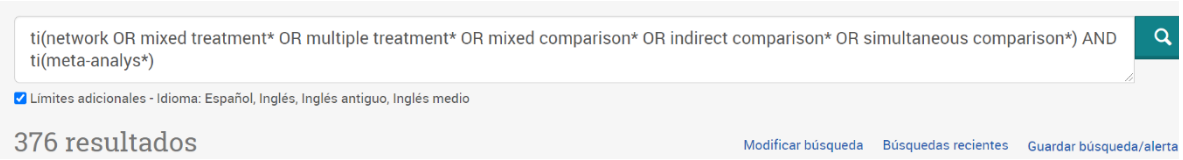

Science Direct

TITLE: (network OR mixed treatment OR multiple treatment OR mixed comparison OR indirect comparison OR simultaneous comparison) AND (meta-analysis)

Filtered by the following subject areas: Social Sciences, Psychology
Filtered by the following research type: Research article OR conference abstract OR review article

ERIC

title:(("network" OR "mixed treatment" OR "multiple treatment" OR "mixed comparison" OR "indirect comparison" OR "simultaneous comparison") AND title:("meta-analysis")

2. Abbreviated search

To avoid language bias, an abbreviated search was done in all databases using the term "Network meta-analysis" translated to five different languages: Spanish (*meta-análisis en red*), German (*meta-analyse AND netzwerk*), Dutch (*meta-analyse AND netwerk*), French (m*éta-analyse AND réseau*) and Chinese (网络元分析 or 网络元分析法). The search was applied to the whole manuscript, that is, it was not restricted to the title as in the original search. In the databases Web of Science, Psycinfo, and Eric, no results were found for any of the searches.

In Scopus, 95 results were found for the Spanish string, of which 93 were not meta-analyses, and two were not network meta-analyses. The French string search led to 73 matches. From these 73 studies, 54 of them were not meta-analyses, 13 were network meta-analyses but published in the field of the medical sciences, three of them were not network meta-analyses, and two studies were not research papers[1]. Finally, in Scopus, three matches were obtained for the German string, but none these three studies were network meta-analysis.

In the database ScienceDirect many matches were obtained: 236 for the Spanish string search, 135 for the French string search, 33 for the German string search, and finally 3 studies for the Dutch string search. From the Spanish matches, 227 were not even meta-analysis, and 9 matches were books or conference proceedings where the term network meta-analysis was not found anywhere. For the search of the French and German string searches, none of the matches were meta-analyses and they were all from fields different from psychology and educational sciences.

In conclusion, no study was retrieved in this abbreviated systematic search.

[1] This abbreviated search was done in January of 2022 after a first round of review. Actually, one network meta-analysis in the field of psychology and educational sciences was found when search the French string in Scopus, but it was published months after the date of the original search of our manuscript (March, 2021), so we did not include it.
